# High exposures to bioactivated cyclophosphamide are related to the occurrence of veno-occlusive disease of the liver following high-dose chemotherapy

**DOI:** 10.1038/sj.bjc.6603097

**Published:** 2006-04-18

**Authors:** M E de Jonge, A D R Huitema, J H Beijnen, S Rodenhuis

**Affiliations:** 1 Department of Pharmacy and Pharmacology, The Netherlands Cancer Institute/Slotervaart Hospital, Louwesweg 6, 1066 EC, Amsterdam, The Netherlands; 2 Department of Medical Oncology, The Netherlands Cancer Institute, Plesmanlaan 121, 1066 CX, Amsterdam, The Netherlands

**Keywords:** bioactivation, correlation, cyclophosphamide, exposure, veno-occlusive disease

## Abstract

We investigated whether the occurrence of veno-occlusive disease of the liver (VOD) may be associated with individual variations in the pharmacokinetics of high-dose cyclophosphamide. Patients received single or multiple courses of cyclophosphamide (1000 or 1500 mg m^−2^ day^−1^), thiotepa (80 or 120 mg m^−2^ day^−1^) and carboplatin (265–400 mg m^−2^ day^−1^) (CTC) for 4 consecutive days. The area under the plasma concentration–time curves (AUCs) were calculated for cyclophosphamide and its activated metabolites 4-hydroxycyclophosphamide and phosphoramide mustard based on multiple blood samples. Possible relationships between the AUCs and the occurrence of VOD were studied. A total of 59 patients (115 courses) were included. Four patients experienced VOD after a second CTC course. The first-course AUC of 4-hydroxycyclophosphamide (*P*=0.003) but not of phosphoramide mustard (*P*=0.101) appeared to be predictive of the occurrence of VOD after multiple courses. High exposures to bioactivated cyclophosphamide may lead to increased organ toxicity.

Cyclophosphamide is an alkylating agent widely used in clinical oncology in both conventional and high-dose chemotherapy regimens. It has been shown that large interindividual variation in clinical effect exist with cyclophosphamide treatment and it has been proposed that these varied responses (both efficacy and toxicity) may reflect interpatient differences in metabolism and distribution of the drug (Huitema *et al*, 2002). However, relationships between the pharmacokinetics of cyclophosphamide and its biological effects are incompletely understood. This is partly explained by the complex metabolic profile of cyclophosphamide (Figure 1) and incomplete understanding of which compounds are involved in clinical response and toxicity.

Cyclophosphamide itself is an inactive prodrug. Approximately 70–80% of the administered drug is bioactivated by cytochrome *P*450 enzymes to form 4-hydroxycyclophosphamide. 4-Hydroxycyclophosphamide is unstable and decomposes into phosphoramide mustard, the ultimate alkylating metabolite. In contrast to 4-hydroxycyclophosphamide, phosphoramide mustard cannot enter target cells, and therefore only the intracellularly formed phosphoramide mustard fraction is considered to be cytotoxic. Plasma concentrations of 4-hydroxycyclophosphamide are therefore a good marker of the alkylating activity of cyclophosphamide (De Jonge *et al*, 2005).

The dose-limiting toxicity of cyclophosphamide at conventional doses is myelosuppression, primarily leukopenia. In the setting of bone marrow transplantation, where higher cyclophosphamide doses can be used, the toxicity profile of cyclophosphamide includes haemorrhagic cystitis, hepatic damage (veno-occlusive disease of the liver (VOD)) pulmonary toxicity and cardiac necrosis (Fraiser *et al*, 1991). Several authors have reported weak relationships between exposure or clearance of cyclophosphamide and its efficacy and toxicity (Ayash *et al*, 1992; Nieto *et al*, 1999; Petros *et al*, 2002; Yule *et al*, 2004). However, few studies have focused on relationships between exposure to cyclophosphamide metabolites and the occurrence of toxicity or efficacy. An indication for a correlation between high exposures to 4-hydroxycyclophosphamide or phosphoramide mustard and the occurrence of VOD was given in three different studies (Honjo *et al*, 1988; Ren and Slattery, 1999; Huitema *et al*, 2002). High exposures to the inactive carboxyphosphamide have also been related to increased incidence of VOD (McDonald *et al*, 2003), which was explained by a positive correlation between 4-hydroxycyclophosphamide and carboxyphosphamide formation.

Veno-occlusive disease of the liver is a life-threatening complication of myeloablative regimens (Shulman *et al*, 1980; Ayash *et al*, 1990; Nevill *et al*, 1991; McDonald *et al*, 1993, 2003). Therefore, we investigated whether a significant relationship could be found between exposures to 4-hydroxycyclophosphamide and phosphoramide mustard and the occurrence of VOD.

## PATIENTS AND METHODS

### Patients and treatment

Patients received the cyclophosphamide, thiotepa and carboplatin (CTC) high-dose chemotherapy regimen with peripheral blood progenitor cell transplantation as described previously (Rodenhuis *et al*, 1996, 1998, 1999, 2003; Schrama *et al*, 2001). Patients had either high-risk primary breast cancer and received high-dose chemotherapy as part of their adjuvant treatment, or had advanced breast, germ-cell or ovarian cancer.

Two different high-dose CTC schedules were used. The full-dose CTC regimen consisted of 4 days of chemotherapy with cyclophosphamide (1500 mg m^−2^ day^−1^) as a 1 h infusion, immediately followed by carboplatin (400 mg m^−2^ day^−1^) as a daily 1 h infusion, and thiotepa (120 mg m^−2^ day^−1^) divided over two 30-min infusions (the second daily dose of TT was administered 12 h after the first dose) (Rodenhuis *et al*, 1998, 2003). The ‘tiny’ CTC regimen (tCTC) was identical to the CTC regimen except that it incorporated 2/3rd of the dose of each agent (Rodenhuis *et al*, 1996, 1999; Schrama *et al*, 2001). Patients received either one (high-risk primary breast cancer) or two (refractory germ-cell cancer) courses of CTC or two (metastatic ovarian cancer) or three (metastatic breast or relapsing germ-cell cancer) courses of tCTC, when possible every 4 weeks. Full details of the CTC and tCTC regimens have been published previously (Rodenhuis *et al*, 1996, 1998, 1999, 2003; Schrama *et al*, 2001).

All protocols were approved by the Committee of Medical Ethics of the Netherlands Cancer Institute and written informed consent was obtained from all patients.

### Sampling and analysis

During the 4-day CTC course, blood samples were collected from a double lumen catheter inserted in a subclavian vein. Complete pharmacokinetic profiles were assessed on two separate days, always including day 1 and either day 3 or 4. Samples were taken at 30 min after the start of CP infusion and at 60 (end of CP infusion), 90, 120 (end of carboplatin infusion), 150 (end of TT infusion), 165, 180, 210, 285, 390 and 660 min. On day 5, an additional sample was collected approximately 22 h after the last CP infusion. A total of approximately 21 samples were available per patient per course. Sample pretreatment and bioanalysis of cyclophosphamide, 4-hydroxycyclophosphamide, phosphoramide mustard, thiotepa, tepa and carboplatin were conducted as described previously (Van Warmerdam *et al*, 1995; Huitema *et al*, 1998, 2000a, 2000b).

### Pharmacokinetic analysis

Pharmacokinetic calculations for cyclophosphamide, 4-hydroxycyclophosphamide and phosphoramide mustard were performed based on two population pharmacokinetic models previously developed in our institute (Huitema *et al*, 2001; De Jonge *et al*, 2004) using the nonlinear mixed effect modelling program NONMEM (double precision, version V 1.1) (Beal and Sheiner, 1998). The applied model is schematically depicted in Figure 2. Parameters were estimated using data of all patients included in the present study. In brief, the model includes both the recognised autoinduction process of cyclophosphamide resulting in increased rate of 4-hydroxycyclophosphamide and phosphoramide mustard formation in time, as well as the inhibitory effect of thiotepa on this induction. For more details on the model we refer to earlier publications (Huitema *et al*, 2001; De Jonge *et al*, 2004).

Based on measured plasma concentrations in the individuals included in this study and the population pharmacokinetic model as presented in Figure 2, Bayesian estimates of the area under the plasma concentration–time curves (AUCs) of 4-hydroxycyclophosphamide and phosphoramide mustard were generated. These estimates were obtained with the POSTHOC option of NONMEM taking both the population pharmacokinetic parameters and the individual data into account (Beal and Sheiner, 1998). AUCs of thiotepa, tepa and carboplatin were calculated in a similar way using previously developed population pharmacokinetic models (Huitema *et al*, 2002; De Jonge *et al*, 2004).

### Clinical diagnosis of VOD

The clinical diagnosis VOD was based on the occurrence of two of the following events within 20 days of transplantation: hyperbilirubinemia (total serum bilirubin >34.2 *μ*mol l^−1^), hepatomegaly (or right upper quadrant pain of liver origin) and ascites (Ayash *et al*, 1990; McDonald *et al*, 1993). The occurrence of VOD was registered in a dichotomous (e.g. no VOD or VOD) way.

### Statistical analysis

Relationships between VOD, occurring after multiple courses, and the cumulative AUCs of 4-hydroxycyclophosphamide and phosphoramide mustard were tested using logistic regression. Also other possible determinants of VOD (exposure to thiotepa and its metabolite tepa, carboplatin exposure or cisplatin pretreatment) were tested in an univariate and subsequently multivariate way. In order to assess whether the AUC of the first course is a prognostic factor for toxicity after subsequent courses, logistic regression was used with both the AUC of the first course and the number of courses administered.

Significance of the logistic regression models was assessed with the Wald statistic. The Statistical Service Solution for Windows version 11.0 (SPSS Inc., Chicago, IL, USA) was used and a two-sided significance level of 0.05 was used for all tests.

## RESULTS

Data were available from a total number of 59 patients (115 courses). In Table 1, the details of the patient population and treatment are summarised. In the first 32 patients (57 courses) pharmacokinetic data were available for cyclophosphamide, 4-hydroxycyclophosphamide, thiotepa, tepa and carboplatin, while in the final 27 patients (58 courses) also data of phosphoramide mustard were available.

A total of four patients experienced VOD. Three patients with refractory germ-cell cancer developed VOD after a second CTC course. One patient, treated for metastatic breast cancer developed VOD after a second tCTC course. Of this latter patient, no phosphoramide mustard data were available for both courses.

Figure 3A and B shows a comparison of the AUC of 4-hydroxycyclophosphamide and phosphoramide mustard during the first course between patients with and without VOD. A significant relationship between the first course AUC of 4-hydroxycyclophosphamide and the occurrence of VOD was found (*P*=0.003) while no significant relationship was found between the first course phosphoramide mustard AUC and VOD (*P*=0.101). Mean AUC of 4-hydroxycyclophosphamide in the first course was 134.5 *μ*M h in the patients without VOD and 184.5 *μ*M h^−1^ in the patients with VOD. For phosphoramide mustard, the mean first course exposure was 526.7 *μ*M h^−1^ in the patients without VOD and 792.0 *μ*M h^−1^ in the patients with VOD. From these results it can be concluded that the exposure to 4-hydroxycyclophosphamide is predictive for hepatic damage after following courses.

No significant relationship was found between the total cumulative 4-hydroxycyclophosphamide or phosphoramide mustard AUC and the occurrence of VOD (*P*=0.173 and 0.428, respectively) in patients receiving two or more courses. Other possible determinants of VOD (exposure to thiotepa and tepa, carboplatin exposure or cisplatin pretreatment) did not result in significant relationships.

## DISCUSSION

The goal of this study was to assess the impact of exposure to bioactivated cyclophosphamide on the occurrence of VOD caused by the high-dose CTC chemotherapy regimen. We found that increased exposures to 4-hydroxycyclophosphamide in course one led to increased occurrence of VOD. Despite the low incidence of VOD (four out of 59 patients developed VOD), statistically significant conclusions could be drawn.

**Figure 1:**
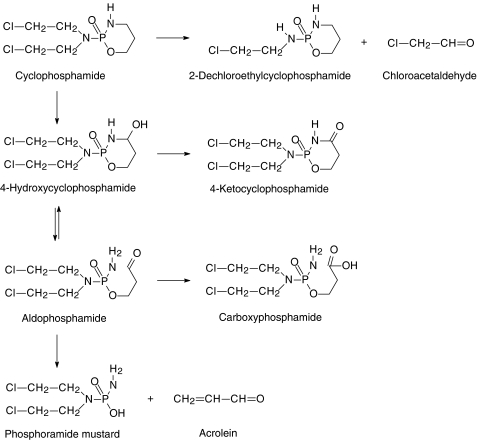
Metabolism of cyclophosphamide, with the bioactivation in the vertical direction and horizontally the inactivation processes.

**Figure 2:**
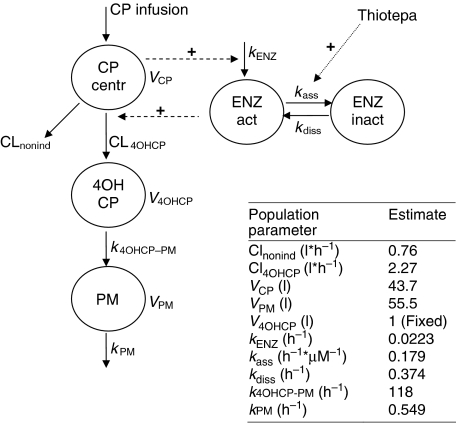
Population pharmacokinetic model of cyclophosphamide (CP) and its metabolites 4-hydroxycyclophosphamide (4OHCP) and phosphoramide mustard (PM).

**Figure 3:**
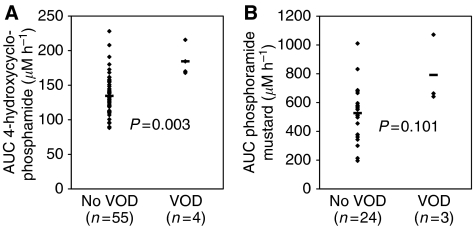
Area under the plasma concentration–time curves (AUCs) of (**A**) 4-hydroxycyclophosphamide and (**B**) phosphoramide mustard of patients with VOD (developed after subsequent courses) *vs* the remaining population, with – being the mean of the data points.

Hepatic injury resulting from treatment with high-dose cyclophosphamide has been reported to be cyclophosphamide dose related (Honjo *et al*, 1988) and may be caused by acrolein which is a by-product in the bioactivation of cyclophosphamide (Figure 1). Acrolein may cause liver dysfunction by binding to cytochrome enzymes, hepatic macromolecules and nucleic acids (DeLeve, 1996). Inhibition of hepatic glutathione-*S*-transferase by acrolein (Scott and Kirsch, 1988), as well as direct glutathione depletion (DeLeve, 1996), may cause acrolein accumulation in the liver after administration of high doses of cyclophosphamide. High-dose busulphan may also cause a depletion of glutathione and glutathione-*S*-transferase levels (Hassan *et al*, 2000). Therefore, the combination cyclophosphamide/busulphan, regularly used in the bone marrow transplantation setting, is often complicated by hepatic toxicity (Nevill *et al*, 1991; McDonald *et al*, 1993).

Our results indicate that the exposure intensity to 4-hydroxycyclophosphamide during treatment may be important in the development of VOD. Higher exposures to 4-hydroxycyclophosphamide in the first course were indicative of the occurrence of VOD later on, possibly because of liver damage not fully recovered before administration of a next similar course (with intrapatient AUC variation between courses of treatment being 23%). Total cumulative exposure to 4-hydroxycyclophosphamide after multiple courses, however, was not significantly correlated, although a trend was observed. This is exemplified by the fact that patients treated with three courses of tCTC received in total the same cyclophosphamide dose as patients receiving two courses of CTC. However, VOD never occurred after 3rd courses of tCTC (in total 19 patients) but occurred in three of the 10 patients receiving two courses of full-dose CTC. Increased risk for VOD in the CTC patients, compared with the tCTC patients, may therefore be explained by administration of a similar total exposure in a shorter time period. From these results it can be concluded that patients receiving a higher exposure intensity to 4-hydroxycyclophosphamide are more likely to develop hepatic damage.

In conclusion, a relationship between the occurrence of VOD and pharmacokinetics of cyclophosphamide has been established. This result indicates that the incidence of VOD may be reduced with lower cyclophosphamide dose intensity or individualised dosing of cyclophosphamide. Therapeutic drug monitoring may be useful for adjusting a cyclophosphamide dose to target the 4-hydroxycyclophosphamide exposure to a value consistent with low toxicity.

## Figures and Tables

**Table 1:** Patients and treatment

**Disease**	**Regimen**	**Number of patients**	**Number of courses**	**Number of 1st courses**	**Number of 2nd courses**	**Number of 3rd courses**
High risk primary breast cancer	1 CTC	16	16	16		
Metastatic breast cancer	3 tCTC	25	63	25	21	17
Refractory germ cell cancer	2 CTC	10	20	10	10	
Refractory germ-cell cancer	3 tCTC	3	8	3	3	2
Metastatic ovarian cancer	2 tCTC	5	8	5	3	
						
Total		59	115	59	37	19

## References

[bib1] Ayash LJ, Hunt M, Antman K, Nadler L, Wheeler C, Takvorian T, Elias A, Antin JH, Greenough T, Eder JP (1990) Hepatic venoocclusive disease in autologous bone marrow transplantation of solid tumors and lymphomas. J Clin Oncol 8: 1699–1706 2213105 10.1200/JCO.1990.8.10.1699

[bib2] Ayash LJ, Wright JE, Tretyakov O, Gonin R, Elias A, Wheeler C, Eder JP, Rosowsky A, Antman K, Frei III E (1992) Cyclophosphamide pharmacokinetics: correlation with cardiac toxicity and tumor response. J Clin Oncol 10: 995–1000 1588381 10.1200/JCO.1992.10.6.995

[bib3] Beal SL, Sheiner LB (1998) NONMEM User's Guides, NONMEM Project Group. San Francisco: University of California at San Francisco

[bib4] De Jonge ME, Huitema ADR, Rodenhuis S, Beijnen JH (2004) Integrated population pharmacokinetic model of both cyclophosphamide and thiotepa suggesting a mutual drug-drug interaction. J Pharmacokin Pharmacodyn 31: 135–156 10.1023/b:jopa.0000034405.03895.c2 15379382

[bib5] De Jonge ME, Huitema ADR, Rodenhuis S, Beijnen JH (2005) Clinical pharmacokinetics of cyclophosphamide. Clin Pharmacokinet 44: 1135–1164 16231966 10.2165/00003088-200544110-00003

[bib6] DeLeve LD (1996) Cellular target of cyclophosphamide toxicity in the murine liver: Role of glutathione and site of metabolic activation. Hepatology 24: 830–837 8855185 10.1002/hep.510240414

[bib7] Fraiser LH, Kanekal S, Kehrer JP (1991) Cyclophosphamide toxicity. Characterizing and avoiding the problem. Drugs 42: 781–795 1723374 10.2165/00003495-199142050-00005

[bib8] Hassan M, Ljungman P, Ringden O, Hassan Z, Oberg G, Nilsson C, Bekassy A, Bielenstein M, Abdel-Rehim M, Georen S, Astner L (2000) The effect of busulphan on the pharmacokinetics of cyclophosphamide and its 4-hydroxy metabolite: time interval influence on therapeutic efficacy and therapy-related toxicity. Bone Marrow Transplant 25: 915–924 10800057 10.1038/sj.bmt.1702377

[bib9] Honjo I, Suou T, Hirayama C (1988) Hepatotoxicity of cyclophosphamide in man: pharmacokinetic analysis. Res Commun Chem Pathol Pharmacol 61: 149–165 3187190

[bib10] Huitema AD, Mathôt RAA, Tibben MM, Rodenhuis S, Beijnen JH (2001) A mechanism-based pharmacokinetic model for the cytochrome P450 drug-drug interaction between cyclophosphamide and thioTEPA and the autoinduction of cyclophosphamide. J Pharmacokinet Pharmacodyn 28: 211–230 11468938 10.1023/a:1011543508731

[bib11] Huitema AD, Spaander M, Mathôt RAA, Tibben MM, Holtkamp MJ, Beijnen JH, Rodenhuis S (2002) Relationship between exposure and toxicity in high-dose chemotherapy with cyclophosphamide, thioTEPA and carboplatin. Ann Oncol 13: 374–384 11996467 10.1093/annonc/mdf052

[bib12] Huitema AD, Tibben MM, Kerbusch T, Zwikker JW, Rodenhuis S, Beijnen JH (1998) Simultaneous determination of *N*,*N*′,*N*″-triethylenethiophosphoramide, cyclophosphamide and some of their metabolites in plasma using capillary gas chromatography. J Chromatogr B, Biomed Sci Appl 716: 177–186 9824231 10.1016/s0378-4347(98)00300-4

[bib13] Huitema ADR, Tibben MM, Kerbusch T, Kettenes-van den Bosch JJ, Rodenhuis S, Beijnen JH (2000a) High performance liquid chromatographic determination of the stabilized cyclophosphamide metabolite 4-hydroxycyclophosphamide in plasma and red blood cells. J Liq Chrom Rel Technol 23: 1725–1744

[bib14] Huitema ADR, Tibben MM, Kerbusch T, Kettenes-van den Bosch JJ, Rodenhuis S, Beijnen JH (2000b) Simple and selective determination of the active cyclophosphamide metabolite phosphoramide mustard in human plasma using high-performance liquid chromatography. J Chromatogr B 745: 345–355 10.1016/s0378-4347(00)00295-4 11043753

[bib15] McDonald GB, Hinds MS, Fisher LD, Schoch HG, Wolford JL, Banaji M, Hardin BJ, Shulman HM, Clift RA (1993) Veno-occlusive disease of the liver and multiorgan failure after bone marrow transplantation: A cohort study of 355 patients. Ann Intern Med 118: 255–267 8420443 10.7326/0003-4819-118-4-199302150-00003

[bib16] McDonald GB, Slattery JT, Bouvier ME, Ren S, Batchelder AL, Kalhorn TF, Schoch HG, Anasetti C, Gooley T (2003) Cyclophosphamide metabolism, liver toxicity, and mortality following hematopoietic stem cell transplantation. Blood 101: 2043–2048 12406916 10.1182/blood-2002-06-1860

[bib17] Nevill TJ, Barnett MJ, Klingemann HG, Reece DE, Shepherd JD, Phillips GL (1991) Regimen-related toxicity of a busulphan-cyclophosphamide conditioning regimen in 70 patients undergoing allogeneic bone marrow transplantation. J Clin Oncol 9: 1224–1232 2045863 10.1200/JCO.1991.9.7.1224

[bib18] Nieto Y, Xu X, Cagnoni PJ, Matthes S, Shpall EJ, Bearman SI, Murphy J, Jones RB (1999) Nonpredictable pharmacokinetic behaviour of high-dose cyclophosphamide in combination with cisplatin and 1,3-bis(2-chloroethyl)-1-nitrosurea. Clin Cancer Res 5: 747–751 10213208

[bib19] Petros WP, Broadwater G, Berry D, Jones RB, Vredenburgh JJ, Gilbert CJ, Gibbs JP, Colvin OM, Peters WP (2002) Association of high-dose cyclophosphamide, cisplatin, and carmustine pharmacokinetics with survival, toxicity, and dosing weight in patients with primary breast cancer. Clin Cancer Res 8: 698–705 11895898

[bib20] Ren S, Slattery JT (1999) Inhibition of carboxyethylphosphoramide mustard formation from 4-hydroxycyclophosphamide by carmustine. AAPS PharmSci 1: E14 11741210 10.1208/ps010314 PMC2761128

[bib21] Rodenhuis S, Bontenbal M, Beex LV, Wagstaff J, Richel DJ, Nooij MA, Voest EE, Hupperets P, van Tinteren H, Peterse HL, TenVergert EM, de Vries EG, Netherlands Working Party on Autologous Transplantation in Solid Tumors (2003) High-dose chemotherapy with hematopoietic stem-cell rescue for high-risk breast cancer. N Engl J Med 349: 7–16 12840087 10.1056/NEJMoa022794

[bib22] Rodenhuis S, de Wit R, de Mulder PHM, Keizer HJ, Sleijfer DT, Lalisang RI, Bakker PJ, Mandjes I, Kooi M, de Vries EG (1999) A multi-center prospective phase II study of high-dose chemotherapy in germ-cell cancer patients relapsing from complete remission. Ann Oncol 10: 1467–1473 10643538 10.1023/a:1008328012040

[bib23] Rodenhuis S, Richel DJ, van der Wall E, Schornagel JH, Baars JW, Koning CC, Peterse JL, Borger JH, Nooijen WJ, Bakx R, Dalesio O, Rutgers E (1998) Randomised trial of high-dose chemotherapy and haemopoietic progenitor-cell support in operable breast cancer with extensive axillary lymph-node involvement. Lancet 352: 515–521 9716055 10.1016/S0140-6736(98)01350-6

[bib24] Rodenhuis S, Westermann A, Holtkamp MJ, Nooijen WJ, Baars JW, van der Wall E, Slaper-Cortenbach IC, Schornagel JH (1996) Feasibility of multiple courses of high-dose cyclophosphamide, thiotepa, and carboplatin for breast cancer or germ cell cancer. J Clin Oncol 14: 1473–1483 8622061 10.1200/JCO.1996.14.5.1473

[bib25] Schrama JG, Baars JW, Holtkamp MJ, Schornagel JH, Beijnen JH, Rodenhuis S (2001) Phase II study of a multi-course high-dose chemotherapy regimen incorporating cyclophosphamide, thiotepa, and carboplatin in stage IV breast cancer. Bone Marrow Transplant 28: 173–180 11509935 10.1038/sj.bmt.1703105

[bib26] Scott TR, Kirsch RE (1988) Inhibition of rat liver glutathione *S*-transferase isoenzymes by acrolein. Biochem Int 16: 439–442 3382415

[bib27] Shulman HM, McDonald GB, Matthews D, Doney KC, Kopecky KJ, Gauvreau JM, Thomas ED (1980) An analysis of hepatic veno-occlusive disease and centrilobular hepatic degeneration following bone marrow transplantation. Gastroenterology 79: 1178–1191 7002704

[bib28] Van Warmerdam LJC, Van Tellingen O, Maes RAA, Beijnen JH (1995) Validated method for the determination of carboplatin in biological fluids by Zeeman atomic absorption spectrometry. Fresenius J Anal Chem 351: 1820–1824 10.1007/s002160000662 11270226

[bib29] Yule SM, Price L, McMahon AD, Pearson AD, Boddy AV (2004) Cyclophosphamide metabolism in children with non-Hodgkin's lymphoma. Clin Cancer Res 10: 455–460 14760065 10.1158/1078-0432.ccr-0844-03

